# Autistic-Like Features in Visually Impaired Children: A Review of Literature and Directions for Future Research

**DOI:** 10.3390/brainsci10080507

**Published:** 2020-08-01

**Authors:** Anna Molinaro, Serena Micheletti, Andrea Rossi, Filippo Gitti, Jessica Galli, Lotfi B. Merabet, Elisa Maria Fazzi

**Affiliations:** 1 Unit of Child Neurology and Psychiatry, ASST Spedali Civili of Brescia, 25121 Brescia, Italy; serena.micheletti@unibs.it (S.M.); andrea.rossi@asst-spedalicivili.it (A.R.); filippo.gitti@asst-spedalicivili.it (F.G.); jessica.galli@unibs.it (J.G.); elisa.fazzi@unibs.it (E.M.F.); 2 Department of Clinical and Experimental Sciences, University of Brescia, 25121 Brescia, Italy; 3 Laboratory for Visual Neuroplasticity, Department of Ophthalmology, Massachusetts Eye and Ear, Harvard Medical School, Boston, MA 02115, USA; lotfi_merabet@meei.harvard.edu

**Keywords:** autism spectrum disorder, autistic-like features, social-cognitive development, stereotypical behaviors, visual impairment

## Abstract

There remains great interest in understanding the relationship between visual impairment (VI) and autism spectrum disorder (ASD) due to the extraordinarily high prevalence of ASD in blind and visually impaired children. The broad variability across individuals and assessment methodologies have made it difficult to understand whether autistic-like symptoms shown by some children with VI might reflect the influence of the visual deficit, or represent a primary neurodevelopmental condition that occurs independently of the VI itself. In the absence of a valid methodology adapted for the visually impaired population, diagnosis of ASD in children with VI is often based on non-objective clinical impression, with inconclusive prevalence data. In this review, we discuss the current state of knowledge and suggest directions for future research.

## 1. Introduction

Research into the presence of autistic-like features among blind children has a long history. Starting from a series of publications appearing in the 1960s and 1970s [1,2,3,4,5,6,7] (which considered that autistic-like behaviors showed by blind children were a possible consequence of the lack of visual experience on the development of self-image and self-representation), researchers and clinicians have increasingly reported commonalities between children with autism spectrum disorder (ASD) and those with visual impairment (VI), particularly with regard to social interaction and communication skills [8,9,10,11,12]. Restricted symbolic play, difficulties in social interaction with peers and imitation, echolalic speech, and increased stereotyped behavior have all been frequently reported in blind children [9,10,13,14]. Indeed, these behaviors resemble subjects with ASD and are often termed “blindisms” since they are explainable in the context of VI [15]. However, the similarity between these “blindisms” and “autistic-like” behaviors, coupled with the lack of ASD assessment tools specifically designed for blind and visually impaired children, complicates the diagnosis of ASD in these individuals. Finally, while the estimated prevalence of ASD among sighted children is between 1 and 2% in Europe [16], determining the prevalence in the visually impaired population still varies greatly, ranging from 2 up to 50% [12,17,18,19]. The underlying mechanisms related to autistic-like symptoms shown by some children with VI, as well as how certain visually impaired children are able to overcome these developmental challenges, remains poorly understood.

Reviewing available literature, it remains to be established whether ASD in VI is primarily a neurodevelopmental condition that occurs independently of the visual disorder (possibly with a common causal agent such as a genetic defect), or is secondary to the VI, and is more closely associated with the disruption of vision on early interactive experiences, or represents a combination of the two [20,21]. For many of the children who are blind and also display features of ASD, it is possible that their characteristics (while being representative of ASD), actually follow a different developmental pathway than those who have ASD and normal vision.

Referring to papers published between 1958 and 2020, the purpose of this review is to provide a discussion of these important, yet still controversial issues. Using two electronic databases (PubMed and Google Scholar), we included combinations of the following search terms: “autism”, “autism spectrum disorder”, “blindness”, “sight loss”, and “visual impairment”. Citations identified from the automated search were manually verified for appropriateness.

The original search yielded 1613 documents, that were reduced to 921 following duplicate removal. Independent screening (by the first and second authors of this review) of the study titles and abstracts was carried out to identify studies that were most relevant to the aims of this review.

Articles were included for further inspection if they satisfied the following inclusion criteria: (1) explored the mechanisms that may explain the observed relationship between VI and ASD, taking into account the nature and role of contributing risk factors such as the severity of VI, type of blindness, age at onset, and other associated impairments; (2) discussed specific behavioral and neurocognitive traits in visually impaired compared to ASD children such as: joint attention, language, verbal and non-verbal communication, theory of mind, stereotypical behaviors; (3) described the approaches employed to assess ASD in visually impaired children, with specific attention given to the fact that the most common methods used for scoring autistic behaviors include several items which are directly dependent on visual abilities; (4) included participants between 0 and 18 years of age.

Articles were included (irrespective of the age range) if they added relevant information, as judged by the authors. Articles were excluded if they were focused on the prevalence and/or the type of ophthalmic problems in the ASD population or the characteristic of visual deficit in specific genetic/metabolic conditions which also presented autistic-like traits. This resulted in the exclusion of 821 papers that did not meet these inclusion criteria and lead to a final sample of 100 studies for the purposes of qualitative synthesis.

## 2. The Observed Relationship between Visual Impairment and ASD: Possible Underlying Mechanisms and Contributing Risk Factors

Since the first reported description by Keeler [22] of a co-occurrence between blindness and autistic behavior, various studies have focused on identifying specific types of ophthalmological disorders as potential organic etiological factors, suggesting the presence of common causal agent potentially independent from the VI itself [22,23,24]. Keeler [22] hypothesized that autistic behavior in children with “retrolental fibroplasia” (i.e., retinopathy of prematurity) resulted from a combination of brain damage, blindness, and emotional deprivation. Wing [25] listed several similarities between children with ASD and children with partial blindness and partial deafness caused by maternal rubella. Chase [24] found a gradient of autistic-like features in a group of 246 individuals with “retrolental fibroplasia”, but none had a clear diagnosis of infantile ASD. The author also reported a strong relationship between autistic-like symptoms and neurological findings. Chess [23] assessed the behavioral data of 243 children with congenital rubella and reported that the common component accounting for ASD in these observed cases was brain damage. Rogers and Newhart-Larson [26] reported the presence of ASD in 5 preschool children with Leber’s congenital amaurosis and compared these children to a control group with congenital blindness due to other causes and typical development, suggesting that cerebellar deficit in some patients with Leber’s congenital amaurosis could provide a neurobiological basis for the behavioral similarities observed between these patients and sighted autistic individuals. Ek and colleagues [27] studied the relationship between blindness due to retinopathy of prematurity (ROP) and ASD and concluded that an ASD diagnosis in blind children is likely to be mediated by brain damage or dysfunction. Fazzi and colleagues [28] submitted 24 children affected by Leber’s congenital amaurosis to a modified version of the Childhood Autism Rating Scale (CARS) by excluding item VII about visual responsiveness [29]. According to their results, 20 children were found to be non-autistic, 4 presented with mild/moderate ASD, and no child was found to be severely autistic. Nearly every child presented some degree of restricted and stereotyped patterns of interest, adherence to specific routines or rituals, difficulties in adapting to environmental changes and showed dysfunctional relationships with other people or in their social and emotional responsiveness. Impaired verbal communication, a tendency for passiveness, and difficulties in using their bodies were also observed. In a prospective study, Garcia-Filion and colleagues [30] demonstrated that autistic-like features occurred with high frequency in children with mild to severe optic nerve hypoplasia (OHN). Since the study included children with various degrees of VI (including those with unilateral ONH), this supported the hypothesis that the autistic component could have a neurological basis, rather than being connected to the visual impairment itself. Similarly, Parr and colleagues [31] assessed the prevalence of social, communicative, and repetitive or restricted behavioral (SCRR) difficulties and defined clinical ASD in 83 children with ONH and/or septo-optic dysplasia (SOD), finding the presence of at least one SCRR difficulty in 58%. Thirty-four percent of the sample was clinically diagnosed with ASD. Moreover, SCRR difficulties and ASD were statistically higher in children with significant cognitive impairment and profound VI and there was no evidence that additional neuro-anatomical abnormalities were a further risk factor in the development of ASD. These data suggested the authors that ASD in children with OHN and/or SOD may arise through different mechanisms compared to the idiopathic ASD population.

Jutley-Neilson and colleagues [32] evaluated the occurrence of ASDs in 28 children with SOD and 14 with ONH. According to the previous study of Parr et al. [31], 33% of children with SOD and ONH received a clinical diagnosis of ASD. Using the Social Communication Questionnaire, 55% of the children met the cut-off threshold for further investigation to differentiate between ASDs and non-ASD (row scores ≥15) and 21% met the cut-off for further investigation to differentiate between ASD and autism (row scores ≥22). The authors identified the degree of visual loss and the severity of intellectual disability as good predictors for ASD, and recommended that children with SOD/ONH would benefit from routine ASDs screening. De Verdier et al. [33] described neurodevelopmental impairments in children with congenital or early infancy blindness born over a decade in Sweden; they found that ASD was one of the most common additional impairment (38% of these population) and that the prevalence was higher in children with ONH (70%), in children with ROP (58%), in children with microphthalmia/anophthalmia (44%), and in children with LCA (36%).

In a different perspective, some researchers [8,10] have suggested that focusing on the cause of blindness is irrelevant, emphasizing rather on the role of sensory deprivation and environmental risk factors in the emergence of autistic-like behaviors. Goodman and Minne [34] assessed 17 congenitally blind children (aged 4 to 11 years) without any additional impairment using the Autism Behavior Checklist [35]. The prevalence of ASD in this sample was 23.5% using a critical cut-off number of symptoms to determine diagnosis. In a study by Brown et al. [36], a prevalence of 20.8% was determined investigating 24 congenitally blind children without any neurological damage (aged 3 to 9 years) using the CARS [29]. Hobson and colleagues [8] found that nine congenitally blind children were similar in their range of clinical features with nine sighted autistic children (age- and verbal IQ-matched).

Regardless of the ophthalmological diagnosis, the potential vulnerability may partially be caused by early blindness and may not only be limited to a lack of vision, but also to severe and early damage to the visual system, threatening the development of mental and emotional processes that allow children to organize experiences and develop different areas of learning [37].

Data from healthy populations suggests that mutual influences between vision and emotion start at very early stages of information processing [38]. The brain regions involved in mental and emotional states include the prefrontal cortex, limbic structures, and the insula as well as visual areas [39]. In particular, enhanced activation of the occipitoparietal regions (corresponding to the dorsal visual processing stream) has been reported during the emotional processing of visual stimuli [39,40]. Abnormal neuronal responses of these cortical regions, such as what could be expected in cerebral visual impairment, may contribute to an impairment in emotional recognition [41].

Recently, Fazzi et al. [19], among 214 children with cerebral causes and 59 with peripheral causes of vision impairment, found that ASD was more prevalent compared to a general population, and that the prevalence varied according to the type of visual disorder (2.8% for cerebral and 8.4% for peripheral visual impairment). Moreover, the presence of autistic symptoms was consistent with the diagnosis of ASD only in subjects with cerebral visual impairment, while in those with peripheral visual impairment, many symptoms related to visual loss overlapped with the clinical features of ASD, making clinical diagnosis more challenging.

Moreover, it is not clear why some children fail to progress, or even regress their communicative and cognitive skills. Mukaddes et al. [17] showed that individuals with blindness and ASD have greater neurological impairment and more severe visual impairment with respect to individuals with blindness only. This suggests that, regardless of the cause of blindness, brain damage remains an important contributing factor for the development of ASD. Certain investigators [42,43] have also described a phenomenon of serious developmental disruption or “setback” which seems to occur between the 15th and 27th month of age. An explanation for this setback occurring in children with profound visual impairment relates to the notion of a sensitive or critical period of brain development within the first to second year of life that relies on normal visual experience occurring within this period [43]. Finally, in a retrospective study by Waugh and colleagues [44], a higher proportion of brain lesions detected with magnetic resonance imaging (MRI) was associated with greater developmental setback in children with visual impairment, which may be an early manifestation of clinical ASD [42,43].

More recently, Vervoled et al. [45] reviewed the literature associated with developmental setback in blind and visually impaired children. Although the authors recognized the period around the second year of life as most vulnerable in these children (particularly in those with neurological abnormalities), they pointed out that the individual variability in development and the wide variability in the methodological aspect make it difficult to draw conclusions on the occurrence of developmental setback in blind and visually impaired children.

It is crucial for professionals who are in contact with these children to recognize these developmental risk signs, namely the presence, persistence, and entrenchment of a whole series of behaviors which are expressions of considerable social isolation. These behaviors include remaining in a lying down position, lack of attention towards environmental stimuli, absence of smiling (or problems eliciting smiling), poor adaptive use of the hands to explore and recognize objects, absent or poor “reach on sound” after the fourth trimester of life, and persistence of excessive and non-functional use of the mouth as the main interface with the environment [34].

## 3. Behavioral and Neurocognitive Traits in Visually Impaired Compared to ASD Children

Although visually impaired children do not present a typical personality profile, it is possible to recognize certain frequently occurring traits, namely high levels of anxiety, some difficulties in social interactions, an excessive production of speech (with declarative rather than communicative intent) serving to fill an emotional void, behavioral rigidities [19], that need to be early detected and constantly monitored. There is a remarkable risk that a blind child’s personality can be limited to body sensations and that the bridge between the self and the outside world can become unstable or even non-existent. If this issue becomes a source of excessive self-restraint, then the onset of problematic behaviors, such as stereotypes, becomes more common in these children [10,46,47]. A presentation of the most representative behavioral and neurocognitive traits that lead to consider the presence of overlapping symptoms between VI and ASD is listed below.

### 3.1. Joint Attention

Sighted babies and young children use visual behaviors like eye contact, gaze following, and joint attention to set up and sustain communication and to learn about the behavior and intentions of others, especially during the pre-linguistic stage [48]. These early visual behaviors and associated interactions appear to lay the foundation for developing emotionally secure attachments, language, and achieving knowledge about self and others [48]. Joint attention is a triadic relationship that arises in the first months of life, based on mutual gaze between the child, an object, and a social partner, in which both the child and the partner are aware of one another’s attention towards an object or event [49]. Visual perception is crucial in this interaction [50].

Joint attention occurs in blind children as well, even if they can acquire it later and differently with respect to sighted children [51]. Infants with VI can be less engaged in joint attention: they usually tend to respond to social interaction with decreased visual attention, pointing [49], or smiling [52]. They are reported to tend to turn head/body away from caregivers and to initiate play interactions with their mothers less often than their sighted peers [13,53]. These behaviors can be interpreted by caregivers as simply a lack of interest, decreasing positive social exchanges [54]. Dale and Salt [48] found that less than a third of the children with profound VI aged 28–40 months were able to share interests and experience with a toy or share interest in an event, in contrast to the great majority (over 80–90%) of the severely visually impaired and sighted children. In a longitudinal study, Urqueta Alfaro and colleagues [54] showed that, in 12-month-old visually impaired infants, the reduction of contrast sensitivity predicted the percentage of time spent in joint engagement. Caregivers of infants/children with VI can learn to interpret and sensibly respond to their baby’s signals through non-visual means [55,56]. Rattray and Zeedyk [57] identified touch, vocalizations, and facial orientation as alternative means to maintain the quality of communicative interactions between mothers and their infants with VI, even if it was not explicitly explained. In their study, infants with VI used active touch during shared attention as a tactile form of communication and made use of facial orientation to a lesser degree than touch and vocalizations, indicating that facial orientation is not as important as an alternative communication means [57].

The atypical development of joint attention in infants with VI, compared to their sighted peers’ developmental patterns, is considered by some authors as a typical sign of ASD [58]. The emergence of joint attention may in fact be disrupted by ASD [59,60]. However, as recently outlined by Urqueta Alfaro et al. [54], the mechanisms and timelines of joint attention development in infants with VI is obviously different from what is expected in infants with typical development, as described above. Failing to recognize this may put VI children at risk of being wrongly labeled as autistic [54]. However, if in ASD the absence/reduction of interest in shared objects and people is a typical feature, as defined in the Diagnostic and Statistical Manual of Mental Disorders (DSM-5) [61], alternative means beyond visual attention is shown in infants with VI to maintain the quality of communicative interactions [62,63].

### 3.2. Language and Communication Skills

Vision is implicated in general language development, as visually driven joint attention experiences in early childhood provide a framework within which language learning occurs [64]. Despite marked variability in visual profiles, children with both peripheral and central VI may exhibit the presence of language and communication disorders [37,64]. This can be a reflection of the visual deficit itself on early interactive experiences, or represent an associated neurodevelopmental condition that occurs independently of the VI or, more frequently a consequence of the two conditions [19].

Communicating with other people can be a challenge both for children with ASD and children with VI, especially as the pragmatic component of language is concerned [64]. As with individuals with ASD [65], children with VI have unique methods of communication (relying instead on non-verbal communication techniques, echolalia, moving from topic to topic, speaking with no eye contact) that may be important in overcoming social barriers.

In children with VI due to central origin, language disorders have been described [37], and may be influenced by both the degree of visual loss and by widespread brain damage that impacts brain network organization and consequently, the development of general neurocognitive functions including language [19].

Language skills have also been widely detailed in children with VI due to peripheral origin and have been considered in the past as the most promising indicator of peripheral VI children’s ability to compensate for early deficits in developing inter-subjectivity [66].

Differently from children with ASD, language may be a developmental domain which provides blind children with alternative non-visual strategies for social development [67] but, similar to children with ASD, adverse outcomes in social communication may be also present in children with both peripheral visual impairment (PVI) and cerebral visual impairment (CVI), probably given to disruptions in visually guided experiences and visual behaviors, which are seen as precursor milestones for subsequent social development [62].

Language includes shared understanding of what words mean (lexicon/semantics); the capacity to change words in systematic ways (morphology); and rules that govern word order in a sentence (syntax). Speech and phonology are the oral means of communicating language. The use of language as a social tool (pragmatics) involves a complex set of rules about using eye contact, interpreting nonverbal messages together with words that may have a different literal meaning. In blind children due to peripheral origin, structural language skills, namely phonology, morphology, and syntax, may allow for fluent conversation and have been described as typically developed, differently from most of the autistic children, in which language impairment is reported [61]. On the other hand, semantic and pragmatic skills, that are required for successful socio-communicative functioning, have been described by Tadic and colleagues [64] as being poorer in both VI and ASD.

Mills [68] outlined that children with VI due to peripheral origin usually develop fully intelligible speech within the same time frame as sighted children. In a recent study, Feng et al. [69] showed that they have enhanced attentional sensitivity to “non-visual” components of language such as phonetic-phonological components. Roder and colleague [70] showed that blind participants were more efficient than sighted children in terms of phonological processing. They score consistently higher than their sighted peers on tests of verbal working memory [71,72,73] as well which, on the contrary, is usually impaired function in children with ASD [74].

With regards to the lexical component, Vinter and colleagues [75] showed that blind children tended to define words denoting concrete animate or inanimate familiar objects evoking their close perceptual experiences of touch, taste, and smell. It was different from what sighted children, who relied their definition on visual perception, and produced more visually oriented verbalism. They also may exhibit atypical conceptual and semantic development [76,77] and demonstrate specific deficits in understanding visual concepts that they have learned through language and not through direct experience. Given fewer opportunities to benefit from traditional classroom education, blind children, due to peripheral disorders, have shown that they may score below their sighted peers on comprehension, similarity, and vocabulary subtest [70,71,78]. Similar to those with ASD [79], young blind children have a limited capacity for generalizing a given word for other items in the category, and use a word for the original referent or only very few items in the category [79].

No significant difficulties with syntactic development have been described in children with PVI [68]. If complexity of structures is analyzed, blind children show similar performance to that of sighted children not only during the first steps of grammatical development, but also taking into account the acquisition of complex sentences [80]. Blind children’s morphological development, with the exception of personal and possessive pronouns usage, has not been described as delayed nor impaired in comparison to the one of sighted children [80]. Dunlea and Andersen [81] have suggested that young blind children use few morphemes such as plural, 3rd person of present indicative, and locative prepositions in organizing structures and imitations. Blind children seem to start to productively use pronouns very late (around age 4), and they produce a great proportion of reversal errors (1st person for 2nd person pronouns and vice versa) [7,81]. On the other hand, language can be delayed in children with CVI [64], whose ability to respond to stimuli has been described by parents as altered [37].

Considering pragmatic aspects, the tendency to use words whose concrete referent is unknown to the speaker, a behavior named verbalism, is another common language behavior of both children with peripheral VI and ASD [82], as is the tendency to use self-oriented language instead of externally oriented language or the tendency to produce a lesser proportion of verbal expressions to offer, show or draw another person’s attention [80].

Echolalia represents one of the peculiar ways of communicating found in children with peripheral VI and ASD. However, learning and using whole phrases or formulas for specific contexts and activities allows to participate in social interactions and share activities with other people [83], while the social role of echolalia in ASD is controversial [84]. Like children with ASD, blind children may ask many questions, sometimes inappropriately, and may make ‘off-the-wall’ comments [83]. They also tend to refer more often to their personal experiences than sighted children when evoking familiar objects [75]. Mothers of children who present severe peripheral VI seem to take more frequent and longer turns at speaking or with other forms of communication than do mothers of sighted children, resulting in an asymmetry between relative dyads’ experiences [67,85]. Parents of blind children also tend to use more response control, more test questions instead of real questions, more requests and more repetitions [56], use more imperatives and requests, and were more likely to introduce the topic of conversation [86] than do mothers of sighted children.

### 3.3. Stereotypical Behaviours

The presence of stereotypical behaviors in children with VI has also been observed and extensively reported in several studies [47,87,88,89,90,91]. Although stereotyped movements are a defining characteristic of ASD, there is also some evidence of a distinct pattern in the visually impaired group. Gal and colleagues [91] assessed self-injurious and other stereotyped movements in children with ASD, vision impairment, intellectual disability, or hearing impairment and in typical children. The group with visual impairment had the second greatest prevalence of manneristic behaviors, but it is also engaged in forms of stereotyped movements sufficiently distinctive and rarely present in other groups. Particularly, visual self-stimulatory behaviors, including eye poking, eye pressing, eye rubbing (which may lead to a number of ocular complications including infections, keratoconus, and corneal scarring), light gazing, and staring, form a large portion of the stereotyped exhibited behaviors by visually impaired children [47,88,92,93,94,95]. These behaviors are generally exclusive to children with VI and are especially present in children with peripheral visual impairment: Jan and colleagues [96] found that those children with a retinal disorder such as Leber’s congenital amaurosis or retinopathy of prematurity were the most intense eye pressers. Other stereotypical behaviors typically observed in visually impaired children are motor stereotypes. These include repetitive head/body rocking, thumb sucking, jumping, swirling, and repetitive hand/finger movements [89,92,93,97,98,99]. However, in a study by McHugh and Lieberman [94], it has been suggested that body rocking often occurs also in children with retinopathy of prematurity and severe VI. This behavior is most likely to occur in those with a CVI, perhaps because of poor motor development in these subjects [97,100]. Similarly, flickering fingers in front of the eyes while staring at light is common in children with CVI and has been interpreted as an extension of light gazing behaviors [101,102].

Various interpretations of stereotyped behaviors have been reported in the literature [92]. For example, some authors considered eye-digital signs as a means to self-stimulate the sensation of light, producing phosphenes (light sensation that result from mechanical pressure on the eyeball that stimulates photoreceptors and activates intact visual pathways) [47]. Other authors have suggested that these behaviors may be caused by an imbalance of neurotransmitters, especially dopamine and serotonin, due to a damage in the central nervous system [100]. Theoretical approaches have been used to explain stereotypical behaviors from a behaviorist, developmental, and functional perspectives [100]. Specifically, children with VI might acquire and maintain stereotyped behaviors because they are reinforced by their consequences (e.g., avoiding an unpleasant situation, or drawing attention), because of a delayed motor development (as an expression of neuromuscular maturation processes), or because these behaviors can act as modulators of arousal state, increasing or decreasing the level of stimulation (e.g., thumb sucking in situations of under-stimulation, repetitive hand movements, and jumping in situation of overstimulation) [91,93,100,103]. According to these hypotheses, the frequency of stereotypic behaviors in visually impaired children seems to decrease with age [93,97] and children affected by isolated visual deficits present stereotyped behaviors which are generally more reversible than the ones found in children with additional disabilities [47]. Further, studies have supported the view that the prevalence and the type of stereotyped behaviors are directly related to the severity of visual impairment [91,92,97]. Early intervention is very important in order to stop stereotyped behaviors from becoming established, entrenched, and irreversible [92]. The purpose of this intervention is to provide support, but also to promote opportunities and situations which will allow children to re-establish contact and communication with the world around them. The way VI impacts children’s development does not solely depend on the sensory limitation itself, but also on the degree caregivers and society accommodate to these children’s needs and strengths [54]. Sensitive parenting in which parents are vocally and tactually responsive to their children’s actions facilitates many blind infants’ ability to learn their interpersonal effectiveness in the social world.

Instead of focusing mainly on visual attention and facial expressions, parents can be encouraged to become more sensitive to their children’s unique inviting signs, pay more attention to the use of movement, touching, tickling, vocalizing, and speech in eliciting physical-tactile and vocal interaction routines [67,104] and to look at body pointing and other unique nonvisual referring signs to create good levels of communication and shared affective meaning about objects and events in the immediate environment [63]. Moreover, the possibility to refer to autobiographical memory is very important in blind children because it is the way they can understand the world. Consequently, unexpected changes in their environment can disturb them and parents should pay attention to guarantee coherence in the personal environment of these children [75].

### 3.4. Theory of Mind

Baron-Cohen [51] has argued that an individual’s eye movements and relationship with a “shared visual attention mechanism” play a key role in establishing a theory of mind module in the developing infant. Hobson [62,105] described foundations of theory of mind and interpersonal understanding in terms of a child taking part in triadic interactions that involve both the child’s and the partner’s awareness of the other’s mutual focus of attention to a third object or event (joint attention). Through joint attention, the child can understand the other person’s attitude towards an object [49], and this behavior is usually carried out via the visual modality [105].

Deficits of theory of mind (ToM) in ASD have been related to a lack of inter-subjectivity in ASD children [106]. In other words, an inability to understand and anticipate the thoughts and emotions of others has been associated with a lack of shared social understanding [107]. Children with VI may have difficulties in understanding thoughts and emotions of others as well since, as Bedny et al. [108] highlighted, congenital blindness can alter two important sources of information that can be considered as building blocks of ToM. At first, it does not permit blind children to learn about other people’s minds via visual observation of other people’s facial expressions or body movements. Secondly, it alters first person experiences of mental life. Specifically, children with VI can understand and share abstract features of other’s experience, but could not have the same experience [108]. It is interesting to note that, differently to individuals with ASD, whose ToM disrumption is debated since the Baron Cohen’s study on 1985 [106], children with congenital VI may present with a delay, but not a deficit in the ToM construction [109], despite not having access to some (visual) information about the mind during development. Eventually, as adults, they can develop a functional and effective ToM, including an understanding of other people’s experience of sight [110].

Evaluating ToM in children with VI can be challenging because many tests used rely on visual capacities. This can help explain 4–7 years delay previously described in developing ToM in congenitally blind children [21,111,112,113,114]. False belief tasks have been particularly used in the evaluation of ToM in children [109]. The first type of false-belief tasks, in which children are expected to predict or explain another agent’s behavior in terms of the agent’s mental states (e.g., Baron-Cohen and colleagues’ “Sally-Anne” task), have been used in assessing ToM in children with VI [113]. Sometimes, they have been based on tasks in which visual experience has a significant role [112,113,114]. Because VI can affect the development of ToM, purely due to visual and perceptual deficits, different tasks from the first-order FB have been needed to evaluate ToM in blind children. Second-order FB tasks were introduced later to examine people’s belief about others’ belief (i.e., “John thinks that Mary thinks that…” [115]), with positive performance provided by children with VI [109,116]. As a matter of fact, in a recent study, the introduction and use of more reliable tools has identified a similar development of ToM capacities in blind children as compared to sighted peers [109]. In Bartoli et al.’s study [109], 17 children with PVI or blindness underwent an adapted version of the ToM Storybooks and performed similarly to the ones of matched typically developing children, matched on chronological age and gender. Pijnacker et al. [116] administered to blind children several first-order and second-order auditory tasks, showing that the visually impaired children’s performances did not differ from sighted children, matched on gender, age, and verbal IQ. These data suggest that the visual nature of the tests or the stimuli should systematically be considered.

Different performance on ToM tasks seem to be related to the type of VI as well. In children with PVI, a delay in ToM development was described in the first studies [21,111,112,113,114], not found in the more recent ones [109,116]. Children with CVI may present a more compromised neurocognitive profile than what is usually expected in children with PVI [117]. Begeer et al. [118] found that ToM performances in children whose blindness involved the optic neural pathways were delayed, compared to the performances of children whose blindness did not involve any neural damage. The detected difficulties in interpreting others’ intentions and reactions that children with CVI showed, could have reflected the deleterious effect of CVI on the understanding of the social context and facial expressions [37]. These difficulties may also be a consequence of the low IQ levels that children with CVI may present [19] and that are in relation to ToM tasks [111]. As suggested by Bartoli et al. [109], a possible future area of research could compare VI children and children with autism matched on verbal IQ, age, and gender, in order to further understand the role of visual experiences on ToM development.

## 4. Methods Used to Assess ASD in Visually Impaired Children

Since there are no consistent results in terms of the relationship between specific types of ophthalmological problems, severity of VI, and the role of associated handicaps (such as hearing deficits, cerebral palsy, epilepsy, and other intellectual disabilities), and their relationship with ASD, it seems necessary to find a new approach when explaining autistic symptoms in the blind and in the sighted population [18]. ASD is known to be highly heterogeneous, and this has made it hard to define a clear phenotype. Although biologically based and with an evident genetic component [119], ASD is defined and diagnosed based on behavioral difficulties, concerning social interaction and the development of communication skills, and repetitive behaviors and restricted interests. Since ASD is defined by a common set of behaviors, it is best represented as a single diagnostic category that is tailored upon the individual’s clinical presentation including clinical characteristics and associated features [120]. Assessing ASD in blind and visually impaired children is a very delicate process in which most of the common methods used to score autistic behavior, including several items linked to vision [121,122] are applied. Therefore, in clinical practice, these standard assessment tools may not be appropriate for specific VI populations [123]. Some authors have designed checklists and/or questionnaires as screening tools to guide further clinical evaluations. Hobson and colleagues [8,20] suggested a checklist containing some clinical features typically found in ASD (derived from DSM-III-R) and used it to interview the children’s teachers. Jutley-Neilson and colleagues [32] used the Social Communication Questionnaire (SCQ), a standardized parent report measure to evaluate communication skills and social functioning in children. Many of the items in the questionnaire involved situations that can only be experienced by sighted children, and the authors highlighted that the SCQ was not as sensitive and specific for visually impaired children. Hoevenaars-van den Boom and colleagues [123] aimed to identify ASD-specific behaviors in deaf-mute people. For this purpose, authors have developed the “observation of characteristics of ASD in persons with deaf-blindness (O-ADB)”, an originally semi-standardized observation tool based on the Autism Diagnostic Observation Schedule [124], the Autism Screening Instrument for Educational Planning [35], the Autism Diagnostic Interview Revised [125], and on the Van Dijk Approach to Assessment [121].

The absence of a valid methodology for this population has often led to the conclusion that diagnosing ASD in children with visual impairment should be based on clinical judgment [122]. However, more recent efforts have been made to adjust or modify the assessment tools used to assist with the clinical diagnosis of ASD in VI children. For example, most authors administer the modified CARS and exclude Item VII on visual responsiveness in order to identify children at risk of developing pervasive developmental disorders [8,20,26,28]

Recently, Williams and colleagues [126] have started applying systematic modifications to the Autism Diagnostic Observation Schedule (ADOS) and the Autism Diagnostic Interview, Revised (ADI-R) in order to assess symptoms of ASD in visually impaired children (the majority of whom have ONH). This pilot study has provided preliminary evidence regarding how to modify ASD measures which are now more useful in the diagnostic evaluation of visually impaired children and both these tools have shown a good agreement with clinical diagnoses. Authors have concluded that additional research is needed to validate the modified measures in larger samples which may include different diagnoses and levels of visual impairment, and also to follow visually impaired children over time to identify common developmental paths and outline whether specific symptoms change over time [126].

In this direction, a recent study by Fazzi et al. [19] employed systematic modifications (i.e., materials and scoring procedures) to the ADOS 2 [127] (second edition) to assess symptoms of ASD in visually impaired children, taking into account the specificity of type of visual disorder (cerebral vs. peripheral visual impairment). In children with CVI, the use of the modified assessment tool (M-ADOS 2) did not modify the diagnostic category, and the clinical diagnosis matched the ADOS 2 classification and the M-ADOS 2 classification in almost all patients. Conversely, among participants with PVI, 16.9% were classified as autism/autism spectrum in accordance to the ADOS-2 scale but only 10% were confirmed using the M-ADOS 2, exhibiting good concordance with the clinical evaluation result. Although preliminary due to the small sample size, the study suggested that autistic-like finding in children with PVI are more influenced by the degree of VI, and specific symptoms may be more reliable than others in discriminating ASD in VI children. The authors point out the importance of using appropriate adapted tools in PVI subjects to avoid overestimation of ASD that may be confounded by the presence of VI and symptoms and habilitation strategies associated with ASD should take into account possible differences in the context of impaired visual abilities.

The utilization of modified assessment tools, specific not only for ASD but also for VI, matched with a careful clinical observation, is needed in order to ensure a correct diagnoses. As clinicians have independently modified existing autism measures to assess children with VI, future challenges associated with improving the diagnostic precision of ASD in VI will be the development of specific assessment based on visual neutral tasks, detailing modifications so that findings can be replicated, and the validation of these tools on larger sample.

## 5. Conclusions

The relationship between VI and ASD is a controversial issue and it is well expressed by the still controversial estimated prevalence of ASD among visually impaired population.

The current review suggests that some evidences can help us in understanding autistic-like behaviors in VI. ASD among visually impaired children can be a neurodevelopmental condition that occurs independently of the visual disorder. This seemed to be particularly true for those described subjects who present potential common causal factors, such as genetic defects, prematurity, pathologies that interest the central nervous system. These conditions cause a combination of blindness and brain damage, which is an important contributing factor for the development of ASD.

Autistic-like symptoms can also be secondary to the VI and related to sensory deprivation and environmental risk factors. This is typical of those children who present only severe VI or blindness, without other disorders that involve the central nervous system. In these cases, the underlying pathway of autistic-like features in VI is distinctive of that of individuals with ASD. Peculiar differences can be found, starting from the great interest in shared objects in blind, but not in ASD individuals; good structural language skills that allow for social participation and shared activities in blind, but not in ASD individuals; evidence of potential reversibility of autistic signs as a transient phenomenon in blind but not in ASD individuals.

According to Brambring [128], in these individuals, autistic-like symptoms may reflect blind-specific developmental problems in the acquisition of social-cognitive abilities rather than a psychopathological disorder. In other words, sighted autistic children and blind children may reveal similar symptoms, but for different reasons.

In visually impaired individuals who present associated problems with potential common causal agent, a detailed analysis of autistic-like symptoms is necessary, in order to avoid an overestimation of the co-occurrence of ASD.

Diagnosing ASD in VI children should be done very carefully in clinical practice and assessment tools that take into account the type and level of VI are needed. The future challenge will be to apply new tests involving alternative nonvisual tasks (e.g., based on tactile or auditory experiences) and to improve our understanding of the alternative developmental pathways and adaptive-compensatory approaches in children with VI and autistic-like symptoms.

## Glossary

ADI-R	Autism Diagnostic Interview, Revised
ADOS	Autism Diagnostic Observation Schedule
ASD	Autism Spectrum Disorder
CARS	Childhood Autism Rating Scale
CVI	Cerebral/Cortical Visual Impairment
DSM-5	Diagnostic and Statistical Manual of Mental Disorders 5th edition
FB	False-Belief task
LCA	Leber Congenital Amaurosis
O-ADB	Observation of characteristics of Autism in persons with Deaf-Blindness
OHN	Optic Nerve Hypoplasia
PVI	Peripheral Visual Impairment
SCQ	Social Communication Questionnaire
SCRR	Social, Communicative, and Repetitive or Restricted behavioral difficulties
SOD	Septo-Optic Dysplasia
ToM	Theory of Mind
VI	Visual Impairment
